# SCGN recruits macrophages by regulating chemokine secretion in clear cell renal cell carcinoma

**DOI:** 10.7150/ijbs.103252

**Published:** 2024-11-04

**Authors:** Tuanjie Guo, Xinchao Zhang, Xuan Wang, Heting Tang, Yang Liu, Siteng Chen, Zhengchuan Niu, Chaofu Wang, Xu Wang, Xiang Wang

**Affiliations:** 1 Department of Urology, Shanghai General Hospital, Shanghai Jiao Tong University School of Medicine, Shanghai, China.; 2 Department of Pathology, College of Basic Medical Sciences, Shanghai Jiao Tong University School of Medicine, Shanghai, China.; 3 Department of Pathology, Ruijin Hospital, Shanghai Jiao Tong University School of Medicine, Shanghai, China.; 4 Department of Urology, Renji Hospital, Shanghai Jiao Tong University School of Medicine, Shanghai, China.; 5 Department of General Surgery, Shanghai Geriatric Medical Center/Zhongshan Hospital, Fudan University, Shanghai, China.

**Keywords:** SCGN, Chemokine, NF-ĸB, Macrophages, ccRCC

## Abstract

Immunotherapy is considered to be one of the most promising curative modalities for cancer, and the effectiveness of immunotherapy depends on the abundance of immune cells in the tumor microenvironment (TME). Immunotherapy tends to be more effective in “hot tumors” characterized by a high abundant immune cells. Our previous studies found that secretagogin (SCGN) showed intranuclear aggregation in the early stages of clear cell renal cell carcinoma (ccRCC) development. However, with tumor progression and distant metastasis of the ccRCC, the expression of SCGN is gradually absent. In this study, we found that SCGN did not affect the malignant phenotype of cancer cells, but could regulate cytokine/chemokine secretion and immune cell migration by performing gene function assays and RNA-seq analyses after overexpressing SCGN in cell lines of ccRCC. Bioinformatics analysis, Transwell and co-culture experiments confirmed that ccRCC cells overexpressing SCGN could recruit M1-type macrophages. Mechanistically, SCGN initiates downstream cytokine/chemokine expression and secretion through the NF-κB signal pathway. This study provides a comprehensive understanding of the function of SCGN in ccRCC. Continuous forced expression of SCGN at different stages may be a potential approach for the treatment of ccRCC.

## Introduction

Renal cell carcinoma (RCC) originates from renal tubular epithelial cells and accounts for 80% of all renal tumors 1. Globally, there are more than 430,000 new cases and 180,000 new deaths each year, with increasing morbidity and mortality rates 2. The incidence of RCC is significantly increased in people over 60 years of age, and two-thirds of the patients are men 3. Clear cell renal cell carcinoma (ccRCC) is the predominant subtype of RCC with the highest incidence of approximately 70% and is the leading cause of RCC-related deaths 4.

There is an almost universal deficiency of most or all of chromosome 3p in hereditary and sporadic ccRCC. A number of genes including *PBRM1*, *SETD2*, *BAP1* and *SETD2* are located on chromosome 3p, and these genes have a relatively high mutation frequency in ccRCC 5. In addition, it is generally accepted that deletion or mutation of the *VHL* gene is one of the mandatory initiating steps in the pathogenesis of ccRCC. The *VHL* gene deletion is inherited in an autosomal dominant manner to the offspring, who usually develop multifocal, bilateral ccRCC 6, 7. Somatic deletion of *VHL* is also observed in most patients with sporadic ccRCC 8. In ccRCC with atypical morphology, the presence of a 3p deletion or a *VHL* mutation is a diagnostic feature of ccRCC. More than 60% of renal cancers are detected incidentally by ultrasound and CT 9. Early-detected ccRCC can be treated with surgery or ablation, but up to one-third of patients will develop metastasis, and ccRCC that develops metastasis is almost always fatal 10. Patients with early-stage limited disease have a high cure rate, with a 5-year survival rate of over 90%. In contrast, the 5-year survival rate for patients with distant metastases rapidly drops to 17% 11.

Since the discovery of immune checkpoint inhibitors and the corresponding basic research, the first-line treatment paradigm for metastatic ccRCC has been updated after trials in multicenter clinical cohorts, especially for intermediate/low-risk patients, with the recent addition of cabozantinib and nivolumab/ipilimumab 11. Macrophages can be mainly classified into M1 and M2-types based on their functions, with M1-types mainly functioning as pro-inflammatory and anti-tumor, whereas M2-types inhibit inflammation and promote tumor progression 12, 13. The immune cells with the highest infiltrating abundance in the TME of ccRCC are macrophages. Univariate analysis showed that high infiltration of CD163^+^ M2-type macrophages was significantly associated with poor clinical prognosis of ccRCC patients 14. Therapeutic strategies targeting macrophages have been focusing on promoting M1-type macrophage infiltration, inhibiting M2-type macrophage recruitment and reprogramming their phenotype from pro-tumorigenic (M2) to anti-tumorigenic (M1). Unfortunately, there have been no major clinical breakthroughs in this approach to date.

In a multicenter clinical cohort study, we demonstrated that ccRCC patients with eosinophilic features had a worse prognosis after dividing them into clear and eosinophilic type groups 15. Simultaneously, analysis of immune infiltration also suggested that there were more M1-type macrophages and monocytes in the immune microenvironment of patients in the clear type group 15. Based on these findings, we explored the molecular heterogeneity between subtypes. We identified a series of gene expression deficiencies in ccRCC patients with eosinophilic features, among which we identified the *SCGN* gene 16. SCGN showed a higher expression level in ccRCC of the clear type, and its expression was completely absent in the eosinophilic type of ccRCC, but the mechanism involved is not clear. Surprisingly, SCGN was not expressed at all in normal renal tissues, showed high expression in the early stage of ccRCC, and with increasing tumor stage grading, the expression level of SCGN was gradually absent, and it was completely absent in metastatic ccRCC 16. In addition, in the same ccRCC patient tumor tissue, when different grading regions were present at the same time, SCGN showed high expression in the region with low malignancy, and SCGN expression was absent in the region with high malignancy 16. This attracts us to further investigate the role of SCGN in ccRCC and its involvement in molecular biological processes.

## Materials and Methods

### Patient cohorts and sample sources

The external datasets used in this study include the TCGA database and the sequencing data of Nilsson et al 5, 17. The specific data extraction methods and processing can be queried in our previous reports 16. The survival analysis cohort came from Ruijin Hospital, and the patient's clinical information could be accessed in our previous study 16. The tissue samples used in this study were obtained from Shanghai General Hospital and were approved by the ethical committee. The study was performed in accordance with the Declaration of Helsinki.

### Cell culture

The origins of HK-2 and RCC4 can be found in our previously published paper 18. 786-0, Caki-1, A498 and THP-1 were purchased from American Type Culture Collection. RCC4 and Caki-1 were cultured in DMEM. 786-O, A498 and THP-1 were cultured in RPMI 1640. HK-2 was cultured in DMEM/F12. All cell lines were supplemented with 10% FBS and 100 U/ml streptomycin/penicillin. All cell lines were examined to ensure that they were free of *mycoplasma* infection. THP-1 culture requires the addition of 1% β-mercaptoethanol (Cat#HY-Y0326, MCE) to prevent polarization. During THP-1 induced process of differentiation, THP-1 monocytes could be differentiated into M0 macrophages by stimulating cells with 200ng/mL PMA (Cat#HY-18739, MCE) for 48 hours. Subsequently, 20 ng/mL IFN-γ (Cat#RP01038, ABclonal) and 100 ng/mL LPS (Cat#HY-D1056, MCE) could make M0 macrophages differentiate to M1-type. In addition, cytokines such as TNFα (Cat#RP00993, ABclonal) were added to the culture systems according to the experimental requirements.

### Real-time quantitative PCR (RT-qPCR)

In this study, all RNA extraction, reverse transcription and PCR were performed using Vazyme kits which are Super FastPure Cell RNA Isolation Kit (Cat#RC102-01), HiScript IV RT SuperMix for qPCR Kit (Cat#R423-01) and ChamQ Universal SYBR qPCR Master Mix Kit (Cat#Q711-02). The specific procedures of the experimental operations were carried out in accordance with the manufacturer's instructions. All primers covered in this study are available in supplementary material Table S1. The Cq values of the target genes were normalized to the housekeeping gene *ACTB*. Fold change was calculated using the 2^-ΔΔCt^ method.

### Western blotting

Simply, cells were prepared by rinsing with ice-cold PBS and lysing in RIPA buffer containing protease and phosphatase inhibitors. Following centrifugation, protein concentration was determined using the BCA Protein Assay Kit, with absorbance measured via a microplate reader. Protein samples were then mixed with SDS loading buffer, boiled, and separated using SDS-PAGE gels before transfer to a PVDF membrane. The membrane was blocked with TBST containing 5% nonfat milk and incubated overnight with primary antibodies at 4 ºC. After washing, secondary antibody incubation occurred at room temperature. Visualization was performed using the Tanon 4600SF Chemiluminescent Image Analysis System. The exact instructions can be found in our previous article 16. The antibodies used were anti-SCGN (Cat# A19615, ABclonal, 1:1000), anti-Vinculin (Cat#201128-T44, SinoBiological, 1:2000), anti-ACTB (Cat#AC038, ABclonal, 1:20000), anti-Vimentin (Cat#A19607, ABclonal, 1:1000), anti-SNAIL (Cat#A11974, ABclonal, 1:1000), anti-N-Cadherin (Cat#A19038, ABclonal, 1:1000), anti-E-Cadherin (Cat#A22333, ABclonal, 1:1000), anti-p-P65 (Cat#A22331, ABclonal, 1:1000), P65 (Cat#A19653, ABclonal, 1:1000), anti-P50(Cat#14220-1-AP, Proteintech, 1:1000) and HRP Goat Anti-Rabbit IgG (Cat#AS014, ABclonal, 1:20000).

### Lentiviral infection and siRNA transfection

SCGN overexpressing lentivirus was purchased from GENECHEM (Shanghai, China). Puromycin was used to screen cell lines for stable transfection. The siRNA was obtained from GenePharma (Shanghai, China), and lipofectamine 2000 (Invitrogen) was used as an auxiliary reagent for transfection of siRNA. The sequence of siRNA can be found in Table S1. The specific steps are as follows: 1) Dilute siRNA into 20 µM storage solution; 2) Dilute 5 µL siRNA with 250 µL Opti-MEM and mix well; 3) Dilute 5 µL lipofectamine 2000 with 250 µL Opti-MEM and mix well, and leave for 5 min at room temperature; 4) Mix siRNA and transfection reagents and mix well, and leave for 20 min at room temperature; 5) Add transfection complex into 6-well cell plate, 500 µL/well and mix well; 6) Place the cell plates in an incubator at 37 °C with 5% CO_2_ overnight and replace the fresh medium. Subsequent experiments were conducted after 48h of lentiviral infection and siRNA transfection of cells.

### Cell Counting Kit-8 (CCK-8) assay

After 48 hours of lentiviral infection or siRNA transfection, 2000 cells per well were inoculated into 96-well plates. Cell proliferation was assessed using a CCK-8 kit (Cat#C0038, Beyotime, China) according to the manufacturer's protocol. The incubation time of all CCK-8 reagents with cells in this study was 1.5h.

### Flow cytometry assay

The Annexin V-PE/7-AAD kit (Cat# 40310ES20, Yeasen) was used to assess the percentage of apoptotic cells. Briefly, 100,000 cells taken 48h after gene intervention were resuspended, centrifuged, and the supernatant discarded. Subsequently, 100 µL of binding buffer was added to gently resuspend the cells, and then 5 µL of Annexin V-PE and 10uL of 7-AAD were added and mixed. The cells were incubated at room temperature protected from light for 15 min and detected by flow cytometry.

### Colony formation assay

Pretreated cells were inoculated in six-well plates with 700 cells per well and incubated for seven days. Methanol fixation and crystal violet staining. The number of colonies was counted and analyzed.

### Transwell assay

Cell invasion was assessed using Transwell chambers. 48 hours after lentiviral infection, 5x10^4 cells were resuspended in 200 µL of serum-free medium and placed in the upper chamber. The bottom of the membrane was pre-coated with Matrigel and 500 µL of complete medium was added to the lower chamber. The cells on the membrane were fixed with methanol for 15 minutes and stained with crystal violet for 15 minutes. Non-invasive cells on the upper side of the membrane were removed, and then migrating cells were counted and photographed under a microscope. Note that the lower chamber in the macrophage migration assay is cancer cells from different treatment groups. The medium used in the macrophage migration experiments was complete medium.

### Single-cell RNA-seq analysis and gene set enrichment analysis

The single-cell RNA-seq data for ccRCC were obtained from the GSE159115 dataset. Quality control, normalization, batch effect correction, identification of highly variable genes, data scaling, PCA and UMAP downscaling were performed sequentially according to the authors' guidelines 19. Gene set enrichment analysis was achieved by GSEA software, and the grouping was based on the median SCGN expression, and differential genes were screened with the parameters |log FC| > 1 and *P* < 0.05 20.

### Immunohistochemistry staining

Paraffin-embedded samples were sliced, deparaffinized by xylene, rehydrated with gradient alcohol, and antigenically repaired in repair solution. The specimens were washed with PBS and blocked by adding serum. The specimens were incubated with primary antibody at 4 °C overnight, and then incubated with secondary antibody at room temperature and protected from light for 1 h. After PBS washing, DAB was added for color development. Hematoxylin was added after PBS washing to re-stain the specimens, which were then rinsed with tap water, dehydrated with gradient alcohol, and then clarified with xylene to seal the slices. Observe and photograph under the microscope.

### RNA-seq and analysis

Total RNA was extracted using TRIzol (Invitrogen) according to the instructions. All RNA samples were evaluated for quantity and purity and sequenced by Majorbio. The analysis was performed on the Majorbio smart cloud platform.

### Immune cells analysis

The analysis of the level of immune cell infiltration was implemented in R by the CIBERSORT algorithm. The specific parameter settings are described in our previously published paper 15.

### Enzyme-linked immunosorbent assay (ELISA)

Cytokines and chemokines were detected in the supernatant or in the cells after 5 days of lentivirus infection of the cells. The assays were performed using commercial kits they were TNFα (Cat#RK00030, ABclonal), CXCL10 (Cat#RK00054, ABclonal), SCGN (Cat#SEK12411, SinoBiological) and the relevant procedures were performed according to the manufacturer's instructions.

### *In vivo* tumor growth assay

All nude mice were purchased from JSJ Laboratory Animal Company (Shanghai, China). 5×10^6^ cancer cells were implanted subcutaneously into 6-week-old male nude mice. Two weeks after tumor transplantation, the mice were euthanized and the tumors were weighed.

### Statistical analysis

The comparison of continuous variables was performed using Wilcoxon test and Student's t-test. Overall survival (OS) was analyzed with Kaplan - Meier curve and compared with log-rank test. All statistical analyses and visualizations were performed in R, Graph Pad Prism, and MedCalc. Differences were considered statistically significant at *P* < 0.05. The ns means no difference; * indicates *P* < 0.05; ** indicates* P* < 0.01; *** indicates* P* < 0.001; and **** indicates* P* < 0.0001.

## Results

### SCGN expression is absent in eosinophilic ccRCC and indicates a poor prognosis

**Figure 1:**
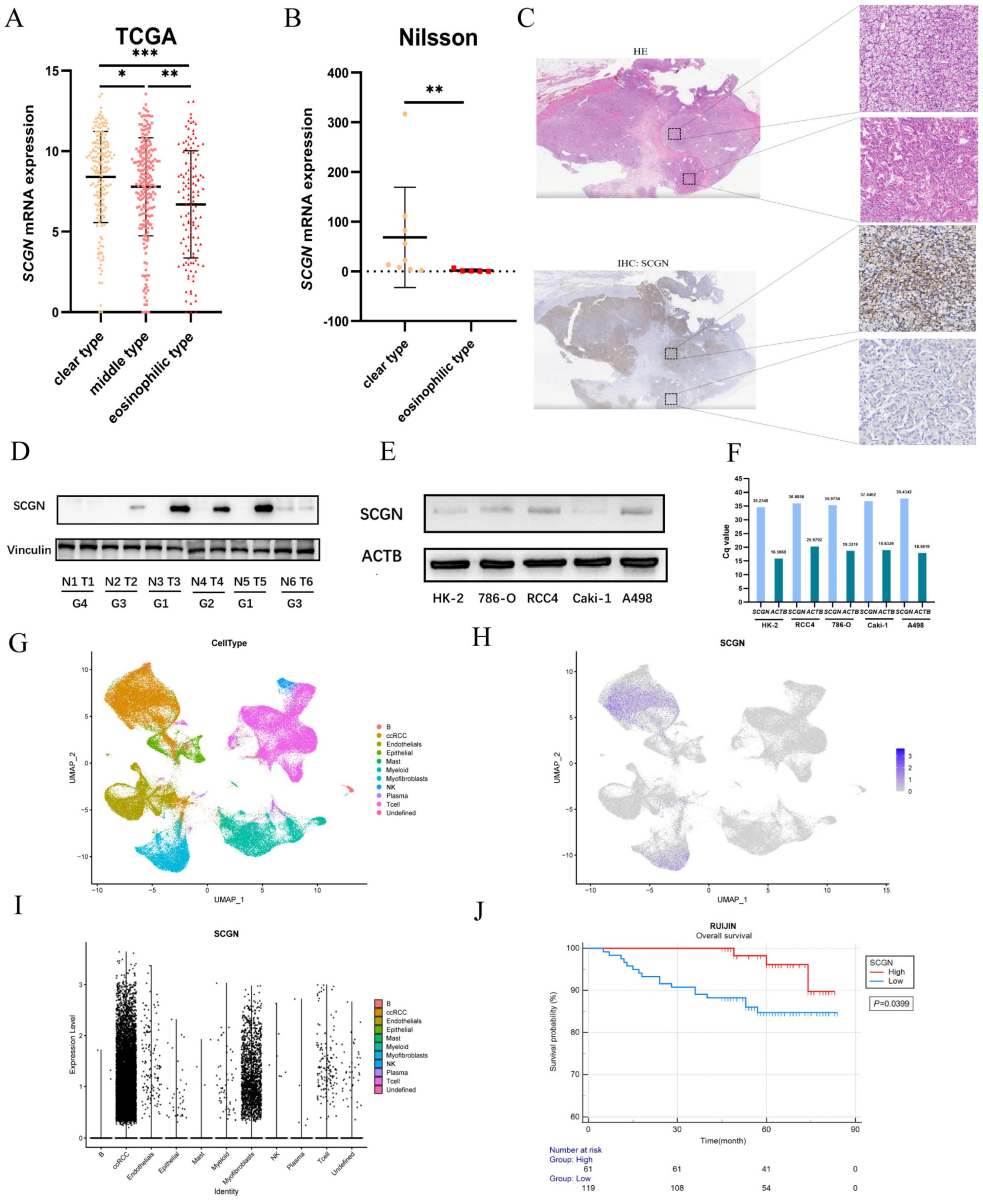
** SCGN expression is heterogeneous and prognostic.** (A) SCGN mRNA expression levels among subtypes in the TCGA database. (B) SCGN mRNA expression levels among different subtypes in the sequencing data of Nilsson et al. (C) Tissue level validation of expression differences between ccRCC subtypes. (D) SCGN expression levels in tumor and adjacent normal tissues. (E-F) SCGN expression levels in different cell lines. (G-I) SCGN expression levels in single cells. (J) Prognostic value of SCGN in our own cohort.

Our previous study found differential expression of SCGN in clear and eosinophilic subtypes but was not validated in a multicenter cohort 15, 16. Through transcriptome sequencing comparison and IHC staining, we found that SCGN is highly expressed in the clear subtype and absent in the eosinophilic subtype, both at the transcriptional and protein levels (Fig. 1A-C). Subsequently, we analyzed SCGN expression in 6 paired ccRCC and adjacent normal tissues as well as in cell lines, and consistently with our previous findings SCGN was highly expressed in tumor tissues, and expression was absent as the nuclear level increased (Fig. 1D). Meanwhile, the results of WB and RT-qPCR showed that SCGN was hardly expressed in the cell lines (Fig. 1E-F). The results of single-cell sequencing also indicated that SCGN was predominantly present in ccRCC cells, but the expression levels varied dramatically from cell to cell, along with significant heterogeneity (Fig. 1G-I). The survival of patients in the tissue microarray stained with SCGN was followed up. We found that the overall survival of patients with high SCGN expression was significantly longer than that of patients with low expression (Fig. 1J). The above findings indicate that the intra-tumoral and inter-tumoral heterogeneity of SCGN has been further validated and the prognostic value of SCGN has been further clarified.

### SCGN has no effect on the malignant phenotype of ccRCC cells

**Figure 2:**
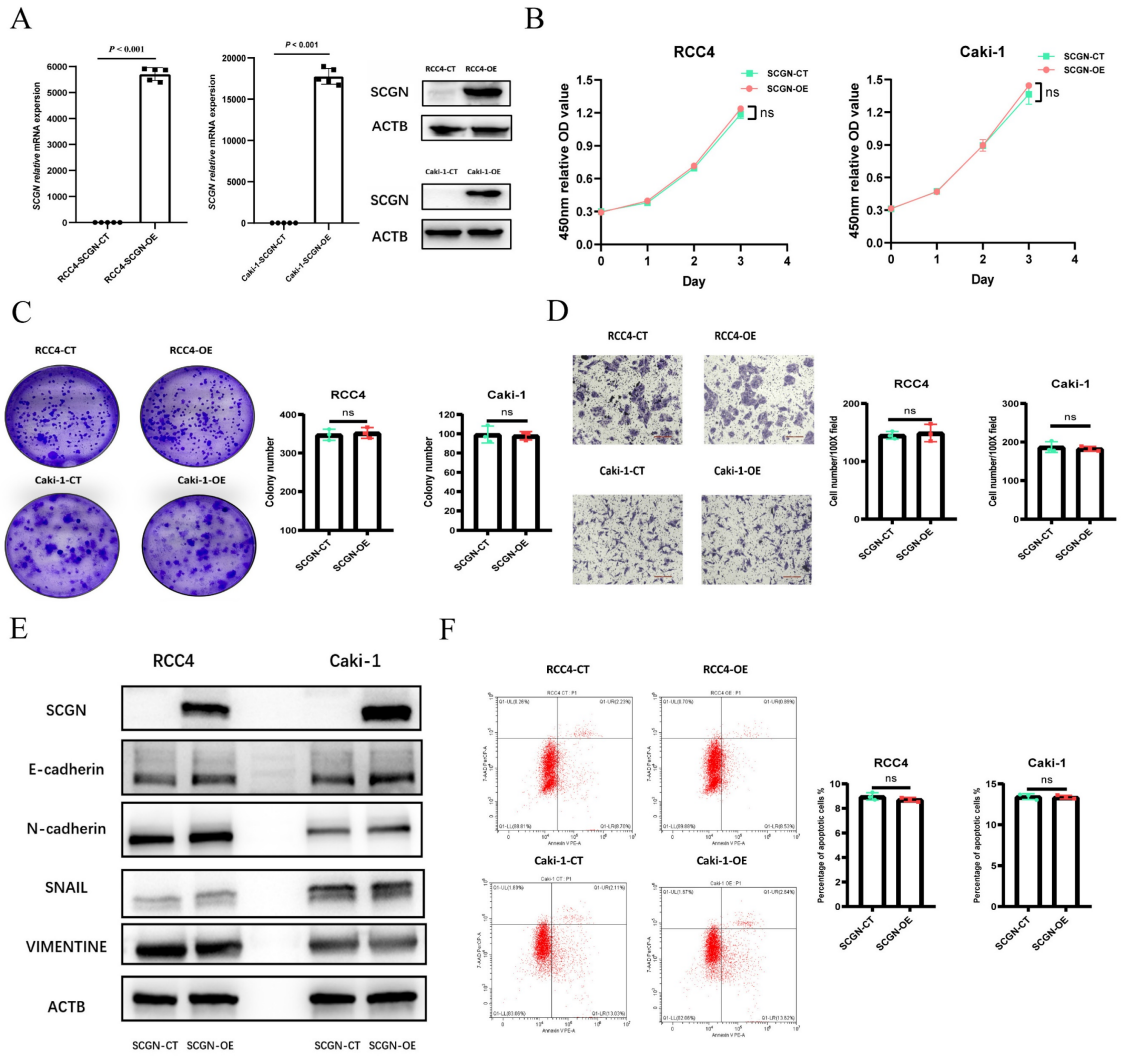
** Overexpression of SCGN does not alter the malignant phenotype of ccRCC cells.** (A) WB and qPCR verified SCGN overexpression. (B) CCK-8 assay after SCGN overexpression. (C) Clone formation assay after SCGN overexpression. (D) Transwell assay after SCGN overexpression. (E) Detection of EMT-related markers after SCGN overexpression. (F) Apoptosis was detected after SCGN overexpression.

Given the significant heterogeneity and lack of expression of SCGN in cell lines, we overexpressed SCGN in cell lines to investigate the function of SCGN. The overexpression results were verified by WB and RT-qPCR (Fig. 2A; Fig. S1A). CCK-8 assay demonstrated that overexpression of SCGN had no effect on the proliferative capacity of the cells (Fig. 2B; Fig. S1B). The results of xenograft tumor experiments indicated that SCGN overexpression also did not affect cancer cell proliferation *in vivo* (Fig. S1C). Apart from that, overexpression of SCGN did not change the clone-forming ability of cancer cells (Fig. 2C). SCGN did not affect the invasive capacity of cancer cells, and the epithelial mesenchymal transition-related markers were not changed (Fig. 2D-E). Some studies have suggested that SCGN may affect apoptosis in cancer cells 21. Therefore, we performed flow cytometry experiments, but we found that SCGN does not change the percentage of apoptotic cells (Fig. 2F). These results suggested that SCGN did not affect the malignant phenotype of cancer cells.

### SCGN is associated with immune cell chemotaxis signaling pathways

**Figure 3:**
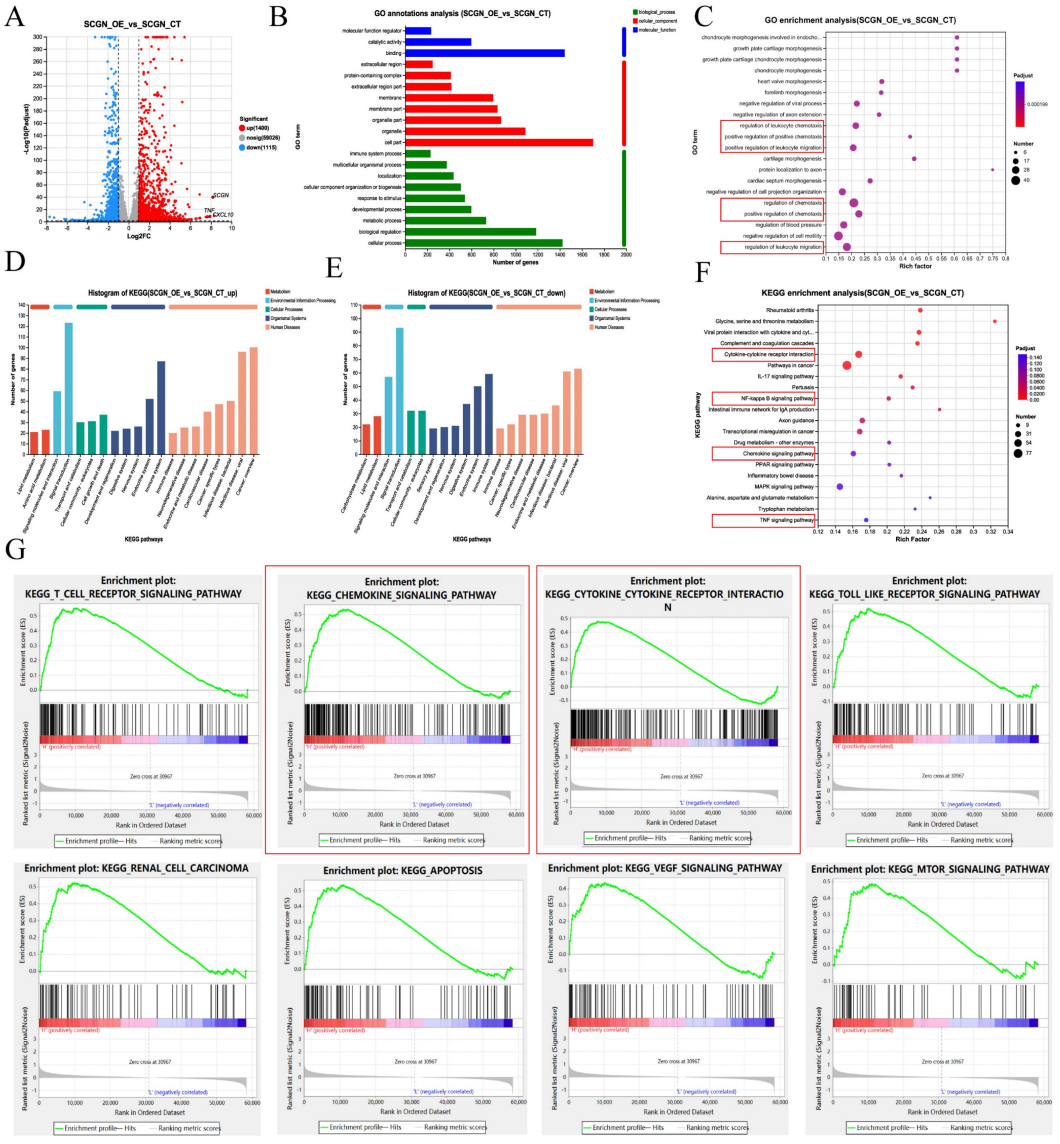
** SCGN is associated with immune cell migration.** (A) Differential gene volcano map between SCGN overexpression and control group. (B) GO annotation analysis of differentially expressed genes. (C) GO enrichment analysis of differentially expressed genes. (D-E) KEGG annotation analysis of differentially expressed genes. (F) KEGG enrichment analysis of differentially expressed genes. (G) KEGG enrichment analysis of differentially expressed genes in the TCGA database.

Given that SCGN does not affect the malignant phenotype of cells and that patients with high SCGN expression have a favorable prognosis, this attracted us to further investigate the underlying mechanisms. The transcriptome was sequenced after overexpression of SCGN on RCC4, among which 1,400 up-regulated and 1,115 down-regulated genes were identified (Fig. 3A). GO annotation and enrichment analysis of differentially expressed genes revealed that SCGN was associated with positive regulation of chemotaxis and positive regulation of leukocyte migration (Fig. 3B-C). KEGG annotation analysis and enrichment analysis of the differentially expressed genes indicated that SCGN regulates cytokine and chemokine signaling pathways, in addition to being associated with NF-ĸB and TNF signaling pathways (Fig. 3D-F). Consistently, analysis of differential genes after grouping based on median SCGN in the TCGA database and subsequent KEGG enrichment analysis using these genes also uncovered that high SCGN expression was associated with cytokines and chemokines (Fig. 3G). Besides that, SCGN was positively correlated with inflammation-related signaling (Fig. S2). Based on these findings we concluded that the better prognosis of patients with high SCGN expression may be attributed to the fact that SCGN regulates chemokine secretion, thereby recruiting immune cells to the TME.

### SCGN regulates M1-type macrophage migration

**Figure 4:**
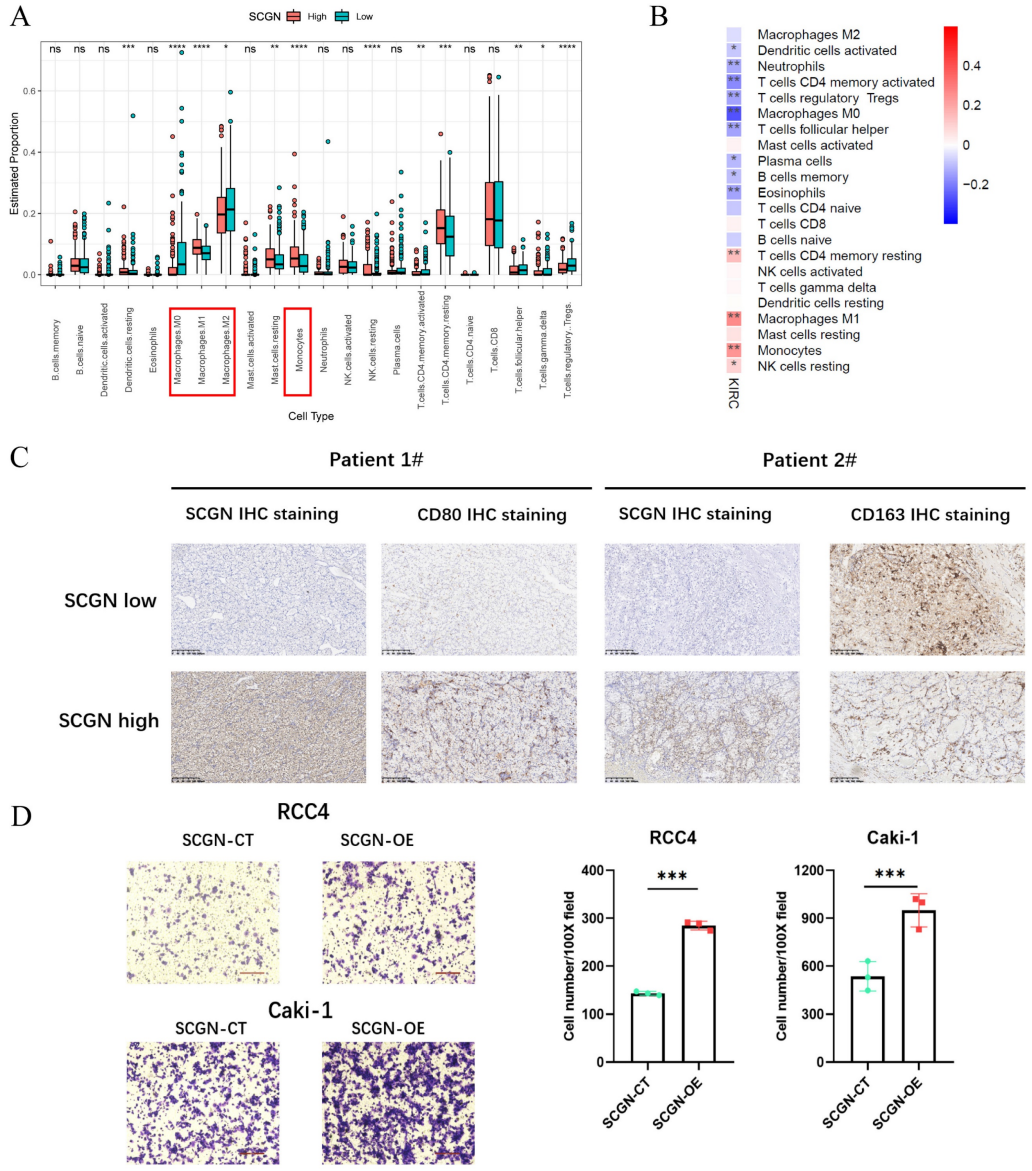
** SCGN regulates M1-type macrophage migration.** (A) Percentage of immune cell infiltration between high and low SCGN expression groups. (B) Correlation analysis of SCGN with immune cells. (C) In paraffin sections stained for SCGN, CD80 and CD163 proteins. (D) *In vitro* cell co-culture detection of M1-type macrophage migration.

According to the results of RNA-seq analysis, SCGN was able to regulate leukocyte chemotaxis. To understand which specific types of cells differed in their percentage between the high and low SCGN groups, we performed an immune cell percentage analysis in the TME. The results of the immune cell proportion analysis indicated that there were multiple cell infiltration levels that differed between the two groups, such as dendritic cells, macrophages, monocytes, NK cells and T-reg cells (Fig. 4A).

There were more M1-type macrophages and monocytes in the SCGN high-expression group and M2-type macrophages in the SCGN low-expression group, which was further confirmed by correlation analysis between SCGN and immune cell levels (Fig. 4B). Markers of M2-type macrophages were significantly upregulated in the SCGN-low group (Fig. S3A). At the tissue level, we further proved that there were more M1-type macrophages in areas of high SCGN expression, whereas there were more M2-type macrophages in areas of low SCGN expression (Fig. 4C). By co-culturing, we also found that SCGN overexpression did not upregulate markers associated with M1 and M2 type macrophages, implying that it does not polarize macrophages (Fig. S3B-C). Besides that, we induced THP-1 cells into M1-type macrophages *in vitro*. Subsequently, co-culture with tumor cells was performed and the number of M1-type macrophages migrating was detected. The results suggested that SCGN overexpressing cancer cells were able to recruit more M1-type macrophages (Fig. 4D). In summary, SCGN was capable of recruiting M1-type macrophages to the TME.

### SCGN recruits M1-type macrophages by upregulating TNFα

**Figure 5:**
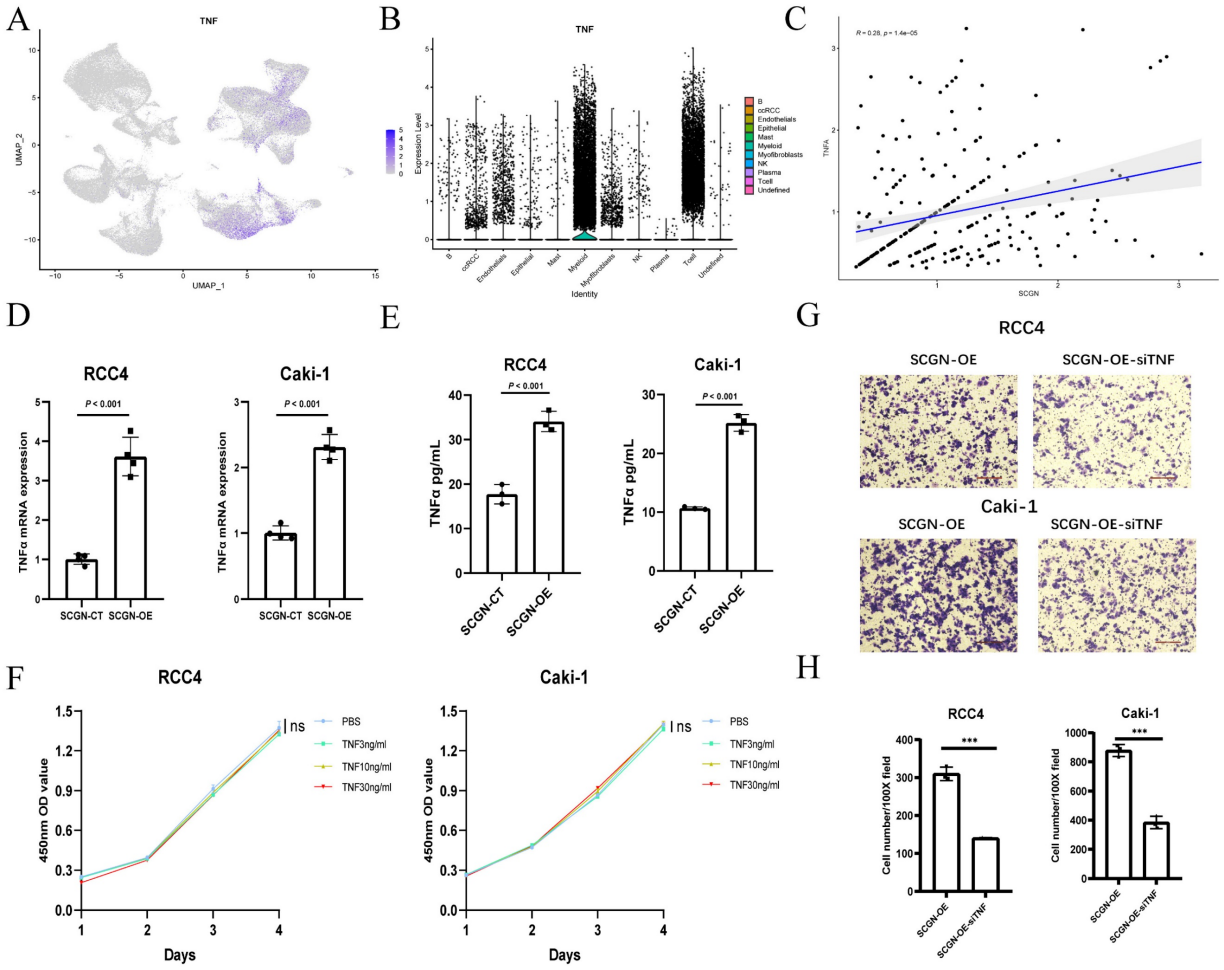
** SCGN recruits M1-type macrophages by upregulating TNFα.** (A-B) *TNF* mRNA expression in various cells in ccRCC. (C) The correlation between *TNF* mRNA and SCGN at the single-cell level. (D-E) Altered expression of TNF at the transcriptional and protein levels after SCGN overexpression. (F) Influence of different concentrations of TNFα on the proliferation of ccRCC cells. (G-H) Migration number of M1-type macrophages after silencing *TNF* mRNA expression.

To explore the potential mechanisms by which SCGN recruits M1-type macrophages into the TME, we reviewed the results of RNA-seq and enrichment analyses, and found that *TNF* mRNA showed significant upregulation after SCGN overexpression. According to previous reports, TNFα can activate T-effector cells (macrophages and NK cells) by blocking T-reg cells, which play an immunosuppressive role, as well as attracting and stimulating and activating neutrophils and monocytes to the tumor site for anti-tumor immune responses 22. Based on the above premise, we analyzed the single-cell data of ccRCC and found that *TNF* mRNA was mainly expressed in myeloid cells and NK cells, but ccRCC cells was also expressed to some extent (Fig. 5A-B). At the single-cell level, the expression of SCGN was positively correlated with *TNF* mRNA (Fig. 5C). According to previous findings, SCGN is highly expressed mainly in the early stage of ccRCC 16. It may be possible that SCGN is highly expressed in the early stage of tumorigenesis and upregulates TNFα to recruit immune cells.

After overexpression of SCGN in ccRCC, we examined the mRNA levels of *TNF* and the concentration of TNFα in the supernatant and found that TNFα was upregulated in both the transcriptome and the supernatant (Fig. 5D-E). Previous reports have shown that TNFα can promote proliferation at low concentrations and apoptosis at high concentrations 23. We detected the effect of different concentrations of TNFα on ccRCC proliferation, and we found that different concentrations of TNFα did not affect the proliferation of ccRCC cells (Fig. 5F; Fig. S4). To demonstrate that TNFα promoted immune cell migration, we performed *in vitro* co-culture experiments, and the M1-type macrophage-promoting migratory effect of SCGN overexpression was partially counteracted when we knocked down *TNF* mRNA in ccRCC (Fig. 5G-H; Fig. S5). These results indicate that SCGN promotes TNFα expression, which in turn regulates M1-type macrophage migration.

### SCGN regulates chemokine secretion via NF-ĸB

**Figure 6:**
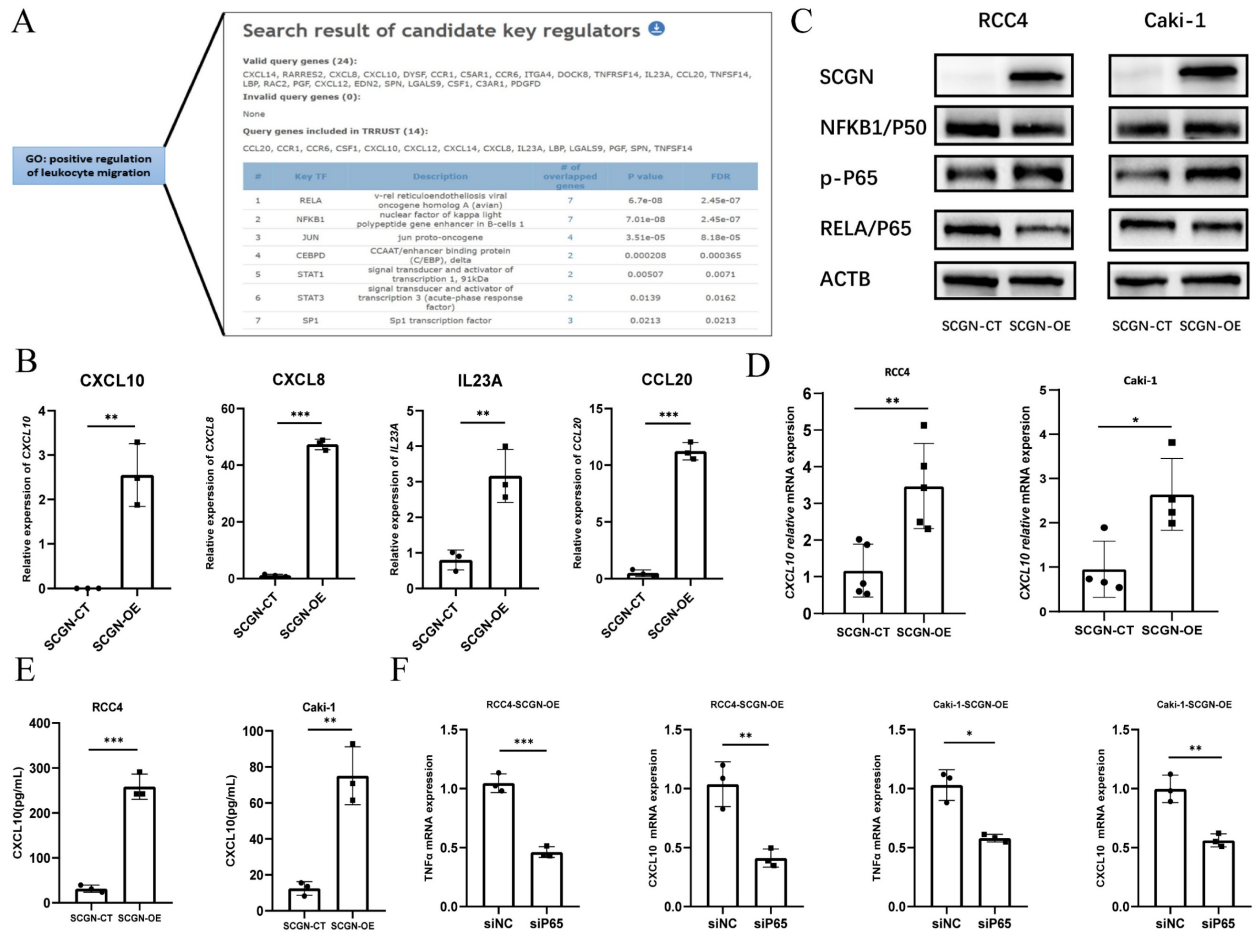
** SCGN regulates chemokine secretion via NF-κB.** (A) Screening of transcription factors by TRRUST (https://www.grnpedia.org/trrust/). (B) Sequencing data for NF-κB-regulated upregulated chemokines. (C) Alteration of NF-κB signaling pathway after SCGN overexpression. (D-E) Verification of transcript and protein levels of CXCL10. (F) Expression of *TNF* and *CXCL10* mRNA after silencing the NF-κB pathway.

**Figure 7:**
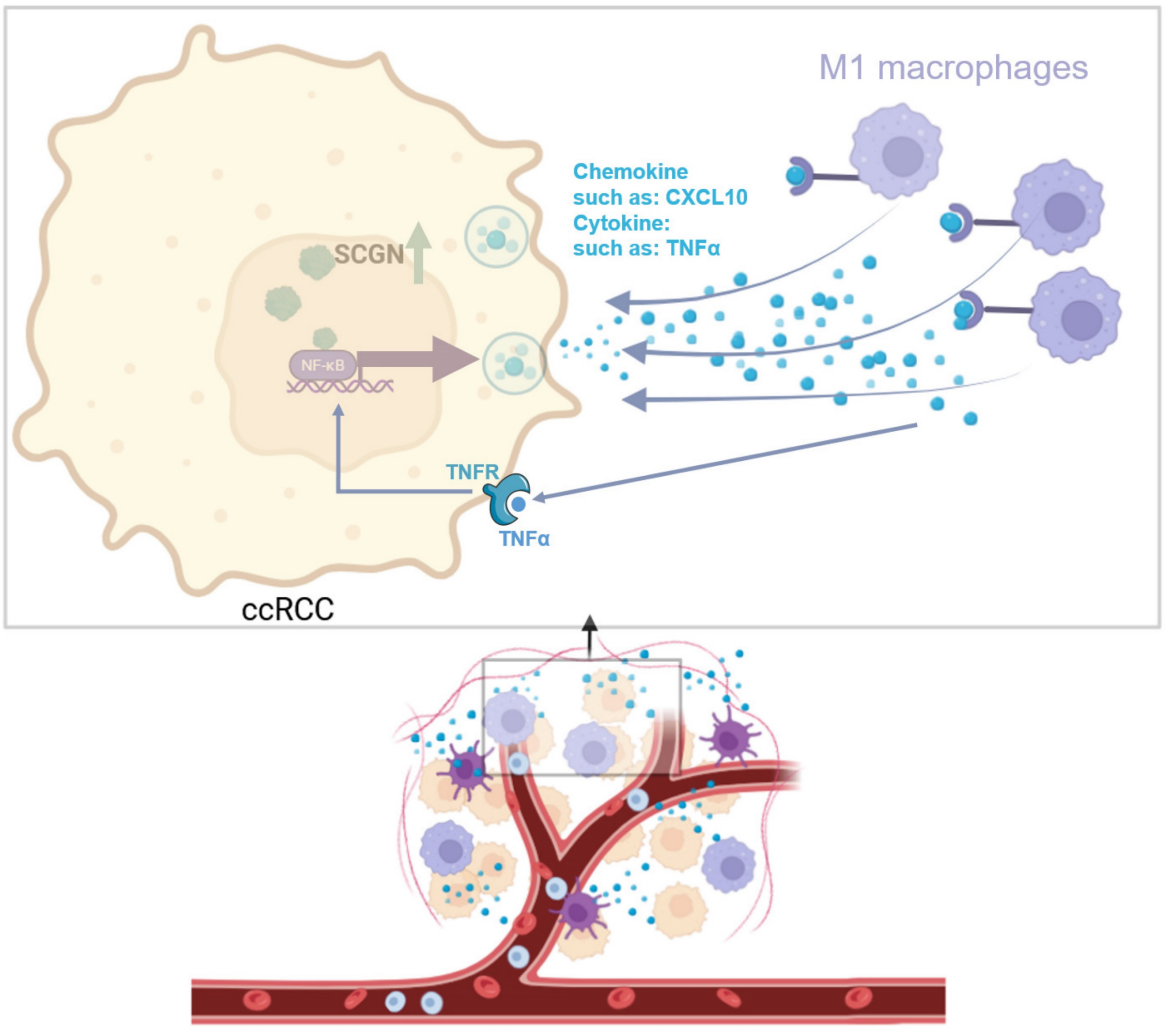
**Mechanism diagram for this study** (image via BioRender).

To clarify the potential mechanism by which SCGN regulates chemokine secretion. We analyzed whether SCGN directly promotes the expression of chemokines by binding to their promoters, but we found that SCGN has no RNA and DNA binding sites. Therefore, we speculated that SCGN promotes chemokine secretion through other signaling pathways. We extracted the genes in the GO enrichment analysis that positively regulated leukocyte migration signaling pathways, and through the TRRUST database we found that the enriched signaling pathway was the NF-ĸB signaling pathway, which regulated the expression of seven chemokines, four of which showed upregulation after SCGN overexpression (Fig. 6A-B). Western blotting confirmed that the NF-ĸB signaling pathway was activated after SCGN overexpression (Fig. 6C). According to previous reports, CXCL10 can act as a chemokine that recruits macrophages and has been linked to prognosis and immunotherapy response 24, 25. We found that after SCGN overexpression, *CXCL10* was upregulated at both transcriptional and protein levels (Fig. 6D-E). When the NF-ĸB pathway was inhibited, both *TNF* and *CXCL10* mRNA expression levels were downregulated (Fig. 6F; Fig. S5; Fig. S6). We therefore conclude that SCGN activates the NF-ĸB pathway and regulates the expression of chemokines to recruit M1-type macrophages. TNFα is also a classic NF-ĸB pathway activating cytokine, and after upregulation of TNFα by SCGN it can further activate the NF-ĸB pathway by acting on tumor cells (Fig. 7).

## Discussion

Intertumoral and intratumoral heterogeneity are typical features of ccRCC. Different patients have distinct TME. In addition, ccRCC is inherently resistant to radiotherapy and chemotherapy, making its treatment options very limited, with only targeted therapy and immunotherapy available to patients. Nonetheless, most patients experience disease progression after targeted therapy and immunotherapy. Meanwhile, not all patients are sensitive to immunotherapy. There is a paucity of markers that reveal tumor heterogeneity and treatment options. In our preliminary study, we found significant differences in patient survival between the eosinophilic and clear subtypes of ccRCC, and identified a new molecular marker that can differentiate the subtypes, i.e., SCGN, and the present study has further confirmed this conclusion 15. Pre-analysis and experiments based on public databases and clinical samples revealed that SCGN was not expressed in normal renal tissues and was highly expressed in tumor tissues, but its expression was gradually absent with tumor progression and metastasis 16. In the TCGA database and our cohort, we found that patients with high SCGN expression had a better prognosis, and with the elevated nuclear grade and stage of ccRCC, the SCGN expression gradually decreased and eventually was completely absent 16. Though SCGN is a secreted protein, it is not secreted extracellularly in ccRCC, therefore we inferred that it functions intracellularly (Fig. S7).

The above findings prompted us to further study the function of SCGN in ccRCC. By detecting the expression level of SCGN in a variety of ccRCC cell lines, we found that the expression level of SCGN in cell lines was very low. This is consistent with speculation, because highly malignant tumors can form cell lines, while SCGN expression is lost as malignant progression progresses. Therefore, we overexpressed the gene in cell lines, and subsequent functional experiments indicated that SCGN did not affect the malignant phenotype of ccRCC cells. Based on these findings, we speculated that SCGN may inhibit tumor progression by affecting the immune system. Therefore, we performed RNA sequencing and bioinformatics analysis of cell lines overexpressing SCGN. We found that SCGN is associated with signaling pathways related to the regulation of immune cell migration. Subsequently, we performed immune cell infiltration analysis. We found that the macrophages with the highest abundance in the TME of ccRCC had significant infiltration differences between the high and low expression groups of SCGN. The abundance of M1 macrophages was high in the high expression group of SCGN, and the abundance of M2 macrophages was high in the low expression group of SCGN, which was also verified at the tissue level. Therefore, we concluded that SCGN was involved in regulating M1-type macrophage infiltration. Mechanistically, we found that one-third of the chemokines involved in signaling pathways regulating immune cell migration were regulated by NF-ĸB, and experiments demonstrated that SCGN activated the NF-ĸB signaling pathway and upregulated TNFα and CXCL10. Silencing TNFα in ccRCC cell lines inhibited M1-type macrophage migration.

Macrophages are the class of immune cells with the highest percentage of infiltration in ccRCC, and several studies have demonstrated that high infiltration levels of M1 inhibit tumor progression, whereas M2-type macrophage infiltration levels have been associated with a poor prognosis in patients with ccRCC 26-30. Originally, we considered that SCGN might have induced macrophage polarization toward M1-type, but the results showed that ccRCC cells overexpressing SCGN co-cultured with M0-type macrophages did not upregulate the markers of M1 macrophages. Ultimately, we concluded that SCGN regulates the migration of M1-type macrophages.

TNFα has different effects in different tumors, which promotes tumor examples in some tumors, inhibits tumor progression in others, and even plays a dual role 31-33. Overall, TNFα is an immunomodulatory factor that activates the immune system and plays a pro-inflammatory role 22, 34. It has been reported that different TNFα concentrations activate different TNFα receptors, which in turn have different subsequent effects. Activation of NF-ĸB induces cell death when activating TNFR1, and activation of endothelial/epithelial cell tyrosine kinase and trans-activates vascular endothelial growth factor receptor 2 promotes cell proliferation when activating TNFR2 23. In our study, we found that SCGN is highly expressed mainly at the early stage of tumorigenesis, and SCGN can upregulate TNFα at an early stage, by NF-ĸB creating an inflammatory TME and thus recruiting M1-type macrophages to inhibit tumor progression.

Canonically NF-ĸB is activated in response to a variety of stimuli, generating rapid but transient transcriptional activity to regulate the expression of various pro-inflammatory genes 35. The NF-ĸB family of transcription factors has been recognized as a central mediator of the inflammatory process and a key participant in both innate and adaptive immune responses 36. Apart from regulating TNFα expression, NF-ĸB also regulates the expression of various chemokines to recruit immune cells 37, 38. In this study, we first reported that SCGN activates NF-ĸB, which regulates the expression of chemokines and TNFα, and that chemokines such as CXCL10 recruit M1-type macrophages, whereas TNFα again activates NF-ĸB to form a loop.

Based on our previous findings that SCGN is specifically highly expressed in tumor tissues in the early stages of ccRCC 16, it could be used as a marker for ccRCC diagnosis, but whether it is specifically present in ccRCC but not in other types of RCC deserves further investigation. If it is expressed only in ccRCC, it will then have the potential to become another specific and vital marker for ccRCC diagnosis. Furthermore, SCGN, as a marker that differentiates clear and eosinophilic features, it is desirable to investigate further whether its absence is directly linked to the appearance of eosinophilic features. Although the present study found that SCGN activated the NF-ĸB signaling pathway, whether it activated the NF-ĸB signaling pathway by up-regulating TNFα or directly was not clear, requiring further studies to elucidate.

One study found that clear types were significantly associated with hypoxia and angiogenesis-related genes compared to eosinophilic types 39. Effector T-cell and immune checkpoint molecule-related genes were elevated to a higher degree in the eosinophilic type 39. In the eosinophilic type cohort, the immune checkpoint treatment group had a significant clinical benefit compared to the tyrosine kinase inhibitor treatment group, and tyrosine kinase inhibitors benefited patients more in the clear type cohort 39. There are currently no validated biomarkers for predicting the efficacy of immunotherapy in ccRCC. Although PD-L1 is overexpressed in approximately 25% of ccRCC tumors and overexpression correlates with poor prognosis, a meta-analysis by Iacovelli and colleagues suggests that its role as a predictive biomarker is unclear 40, 41. With nivolumab/ipilimumab, there was a trend towards a better prognosis for patients with tumor specimen PD-L1 expression ≥1% in CheckMate 214 42, but this trend was not evident for nivolumab in CheckMate 025 43. Since SCGN can be used as a marker to differentiate subtypes, exploring whether SCGN can predict immunotherapy efficacy also warrants further work. The present study also found that SCGN may be a key target for the regulation of macrophage infiltration, and its upstream genes that inhibit its transcription may be key factors for the inhibition of macrophage infiltration. Combined immunotherapy for its inhibition may play a role in enhancing immunotherapy. Apart from that, given that the SCGN low-expression group had more M2-type macrophage infiltration, this implies that SCGN low-expression patients may benefit from anti-PD-1/PD-L1 therapy, but this requires further studies to be clarified.

While this study identified potential mechanisms by which SCGN inhibits the progression of ccRCC, this study did not delve into the specific mechanisms by which SCGN activates the NF-κB pathway. Given the lack of murine-derived cell lines for ccRCC, we only stained clinical samples from ccRCC patients for macrophage typing and did not transplant tumors to analyze the proportion of immune cell infiltration in ccRCC cell lines after SCGN overexpression. This also requires further experiments to confirm.

## Conclusions

In this study, we found that SCGN can serve as a biomarker for the development and progression of ccRCC, and a better prognosis was demonstrated for patients with high SCGN expression at both the mRNA and protein levels. There were differences in SCGN expression in tumors of patients with different nuclear grades. Meanwhile, it was demonstrated that SCGN expression did not affect the malignant phenotype of cancer cells, but inhibited cancer progression by recruiting M1-type macrophages through secreting cytokines/chemokines. We also clarified that SCGN secretes cytokines/chemokines by regulating the NF-κB signaling pathway, identified specific chemokines that recruit M1-type macrophages, and validated the signaling pathways that regulate cytokine/chemokine secretion. This study defined the role of SCGN in ccRCC and demonstrated that SCGN may be a key factor in regulating M1-type macrophage infiltration.

## Supplementary Material

Supplementary **Table S1:** Primers and siRNA sequences. **Figure S1:** SCGN did not affect the proliferation of ccRCC cells. **Figure S2:** SCGN and inflammation signaling are strongly correlated.** Figure S3:** SCGN regulates macrophage infiltration but does not influence polarization. **Figure S4:** TNF did not affect ccRCC proliferation.** Figure S5:** siRNA knockout inefficiency validation.** Figure S6:** Inhibition of NF-κB reduces cytokine and chemokine expression. **Figure S7:** SCGN concentrations in cell lysates and supernatants.

## Abbreviations

RCC: renal cell carcinoma
ccRCC: clear cell renal cell carcinoma
SCGN: secretagogin
TCGA: The Cancer Genome Atlas
TME: tumor microenvironment
RT-qPCR: Real-time quantitative PCR. ELISA: Enzyme-linked immunosorbent assay
OS: overall survival
CCK-8: Cell counting kit-8
KEGG: Kyoto Encyclopedia of Genes and Genomes
GO: Genetic Ontology
GSEA: gene set enrichment analysis

## References

[1] Singh D (2021). Current updates and future perspectives on the management of renal cell carcinoma. Life sciences.

[2] Sung H, Ferlay J, Siegel RL, Laversanne M, Soerjomataram I, Jemal A (2021). Global Cancer Statistics 2020: GLOBOCAN Estimates of Incidence and Mortality Worldwide for 36 Cancers in 185 Countries. CA: a cancer journal for clinicians.

[3] Jonasch E, Walker CL, Rathmell WK (2021). Clear cell renal cell carcinoma ontogeny and mechanisms of lethality. Nat Rev Nephrol.

[4] Barata PC, Rini BI (2017). Treatment of renal cell carcinoma: Current status and future directions. CA: a cancer journal for clinicians.

[5] Cancer Genome Atlas Research Network (2013). Comprehensive molecular characterization of clear cell renal cell carcinoma. Nature.

[6] Latif F, Tory K, Gnarra J, Yao M, Duh FM, Orcutt ML (1993). Identification of the von Hippel-Lindau disease tumor suppressor gene. Science (New York, NY).

[7] Lonser RR, Glenn GM, Walther M, Chew EY, Libutti SK, Linehan WM (2003). von Hippel-Lindau disease. Lancet (London, England).

[8] Nickerson ML, Jaeger E, Shi Y, Durocher JA, Mahurkar S, Zaridze D (2008). Improved identification of von Hippel-Lindau gene alterations in clear cell renal tumors. Clinical cancer research: an official journal of the American Association for Cancer Research.

[9] Petejova N, Martinek A (2016). Renal cell carcinoma: Review of etiology, pathophysiology and risk factors. Biomedical papers of the Medical Faculty of the University Palacky, Olomouc, Czechoslovakia.

[10] Jonasch E, Gao J, Rathmell WK (2014). Renal cell carcinoma. BMJ (Clinical research ed).

[11] Atkins MB, Tannir NM (2018). Current and emerging therapies for first-line treatment of metastatic clear cell renal cell carcinoma. Cancer treatment reviews.

[12] Colegio OR, Chu NQ, Szabo AL, Chu T, Rhebergen AM, Jairam V (2014). Functional polarization of tumour-associated macrophages by tumour-derived lactic acid. Nature.

[13] Nasir I, McGuinness C, Poh AR, Ernst M, Darcy PK, Britt KL (2023). Tumor macrophage functional heterogeneity can inform the development of novel cancer therapies. Trends in immunology.

[14] Komohara Y, Hasita H, Ohnishi K, Fujiwara Y, Suzu S, Eto M (2011). Macrophage infiltration and its prognostic relevance in clear cell renal cell carcinoma. Cancer Sci.

[15] Guo T, Jiang L, Wang T, Zhang J, Liu Y, Wang X (2023). Screening and identification of prognostic genes associated with eosinophilic features of clear cell renal cell carcinoma. Heliyon.

[16] Guo T, Wang X, Wang T, Zhang J, Liu Y, Chen S (2024). Dynamic changes of SCGN expression imply different phases of clear cell renal cell carcinoma progression. Discov Oncol.

[17] Nilsson H, Lindgren D, Axelson H, Brueffer C, Saal LH, Lundgren J (2020). Features of increased malignancy in eosinophilic clear cell renal cell carcinoma. J Pathol.

[18] Zhang X, Li S, He J, Jin Y, Zhang R, Dong W (2022). TET2 Suppresses VHL Deficiency-Driven Clear Cell Renal Cell Carcinoma by Inhibiting HIF Signaling. Cancer research.

[19] Zhang Y, Narayanan SP, Mannan R, Raskind G, Wang X, Vats P (2021). Single-cell analyses of renal cell cancers reveal insights into tumor microenvironment, cell of origin, and therapy response. Proc Natl Acad Sci U S A.

[20] Subramanian A, Tamayo P, Mootha VK, Mukherjee S, Ebert BL, Gillette MA (2005). Gene set enrichment analysis: a knowledge-based approach for interpreting genome-wide expression profiles. Proceedings of the National Academy of Sciences of the United States of America.

[21] Yang X-Y, Liu Q-R, Wu L-M, Zheng X-L, Ma C, Na R-S (2018). Overexpression of secretagogin promotes cell apoptosis and inhibits migration and invasion of human SW480 human colorectal cancer cells. Biomed Pharmacother.

[22] Josephs SF, Ichim TE, Prince SM, Kesari S, Marincola FM, Escobedo AR (2018). Unleashing endogenous TNF-alpha as a cancer immunotherapeutic. Journal of translational medicine.

[23] Al-Lamki RS, Sadler TJ, Wang J, Reid MJ, Warren AY, Movassagh M (2010). Tumor necrosis factor receptor expression and signaling in renal cell carcinoma. The American journal of pathology.

[24] Tokunaga R, Zhang W, Naseem M, Puccini A, Berger MD, Soni S (2018). CXCL9, CXCL10, CXCL11/CXCR3 axis for immune activation - A target for novel cancer therapy. Cancer Treat Rev.

[25] Reschke R, Yu J, Flood B, Higgs EF, Hatogai K, Gajewski TF (2021). Immune cell and tumor cell-derived CXCL10 is indicative of immunotherapy response in metastatic melanoma. J Immunother Cancer.

[26] Díaz-Montero CM, Rini BI, Finke JH (2020). The immunology of renal cell carcinoma. Nature reviews Nephrology.

[27] Xu Y, Li L, Yang W, Zhang K, Zhang Z, Yu C (2023). TRAF2 promotes M2-polarized tumor-associated macrophage infiltration, angiogenesis and cancer progression by inhibiting autophagy in clear cell renal cell carcinoma. Journal of experimental & clinical cancer research: CR.

[28] Xie Y, Chen Z, Zhong Q, Zheng Z, Chen Y, Shangguan W (2021). M2 macrophages secrete CXCL13 to promote renal cell carcinoma migration, invasion, and EMT. Cancer cell international.

[29] Chen S, Qian S, Zhang L, Pan X, Qu F, Yu Y (2022). Tumor-associated macrophages promote migration and invasion via modulating IL-6/STAT3 signaling in renal cell carcinoma. International immunopharmacology.

[30] Fu Q, Xu L, Wang Y, Jiang Q, Liu Z, Zhang J (2019). Tumor-associated Macrophage-derived Interleukin-23 Interlinks Kidney Cancer Glutamine Addiction with Immune Evasion. European urology.

[31] Balkwill F (2009). Tumour necrosis factor and cancer. Nature reviews Cancer.

[32] Balkwill F (2006). TNF-alpha in promotion and progression of cancer. Cancer metastasis reviews.

[33] Cruceriu D, Baldasici O, Balacescu O, Berindan-Neagoe I (2020). The dual role of tumor necrosis factor-alpha (TNF-α) in breast cancer: molecular insights and therapeutic approaches. Cellular oncology (Dordrecht, Netherlands).

[34] Laha D, Grant R, Mishra P, Nilubol N (2021). The Role of Tumor Necrosis Factor in Manipulating the Immunological Response of Tumor Microenvironment. Front Immunol.

[35] Yu H, Lin L, Zhang Z, Zhang H, Hu H (2020). Targeting NF-κB pathway for the therapy of diseases: mechanism and clinical study. Signal transduction and targeted therapy.

[36] DiDonato JA, Mercurio F, Karin M (2012). NF-κB and the link between inflammation and cancer. Immunological reviews.

[37] Fan Y, Mao R, Yang J (2013). NF-κB and STAT3 signaling pathways collaboratively link inflammation to cancer. Protein & cell.

[38] Korbecki J, Kojder K, Kapczuk P, Kupnicka P, Gawrońska-Szklarz B, Gutowska I (2021). The Effect of Hypoxia on the Expression of CXC Chemokines and CXC Chemokine Receptors-A Review of Literature. International journal of molecular sciences.

[39] Yoshida T, Ohe C, Ikeda J, Atsumi N, Ohsugi H, Sugi M (2021). Eosinophilic features in clear cell renal cell carcinoma correlate with outcomes of immune checkpoint and angiogenesis blockade. J Immunother Cancer.

[40] Rijnders M, de Wit R, Boormans JL, Lolkema MPJ, van der Veldt AAM (2017). Systematic Review of Immune Checkpoint Inhibition in Urological Cancers. European urology.

[41] Iacovelli R, Nolè F, Verri E, Renne G, Paglino C, Santoni M (2016). Prognostic Role of PD-L1 Expression in Renal Cell Carcinoma. A Systematic Review and Meta-Analysis. Targeted Oncology.

[42] Motzer RJ, Tannir NM, McDermott DF, Arén Frontera O, Melichar B, Choueiri TK (2018). Nivolumab plus Ipilimumab versus Sunitinib in Advanced Renal-Cell Carcinoma. The New England journal of medicine.

[43] Motzer RJ, Escudier B, McDermott DF, George S, Hammers HJ, Srinivas S (2015). Nivolumab versus Everolimus in Advanced Renal-Cell Carcinoma. The New England journal of medicine.

