# Supplementary material for: SCGN recruits macrophages by regulating chemokine secretion in clear cell renal cell carcinoma
Source: Int J Biol Sci. 2024 Nov 4;20(15):5925–38. doi: 10.7150/ijbs.103252 (PMC11628334; doi:10.7150/ijbs.103252)
Supplement: Supplementary file 1 — Supplementary Table S1: Primers and siRNA sequences. Figure S1: SCGN did not affect the proliferation of ccRCC cells. Figure S2: SCGN and inflammation signaling are strongly correlated. Figure S3: SCGN regulates macrophage infiltration but does not influence polarization. Figure S4: TNF did not affect ccRCC proliferation. Figure S5: siRNA knockout inefficiency validation. Figure S6: Inhibition of NF-κB reduces cytokine and chemokine expression. Figure S7: SCGN concentrations in cell lysates and supernatants. [file ijbsv20p5925s1.pdf]

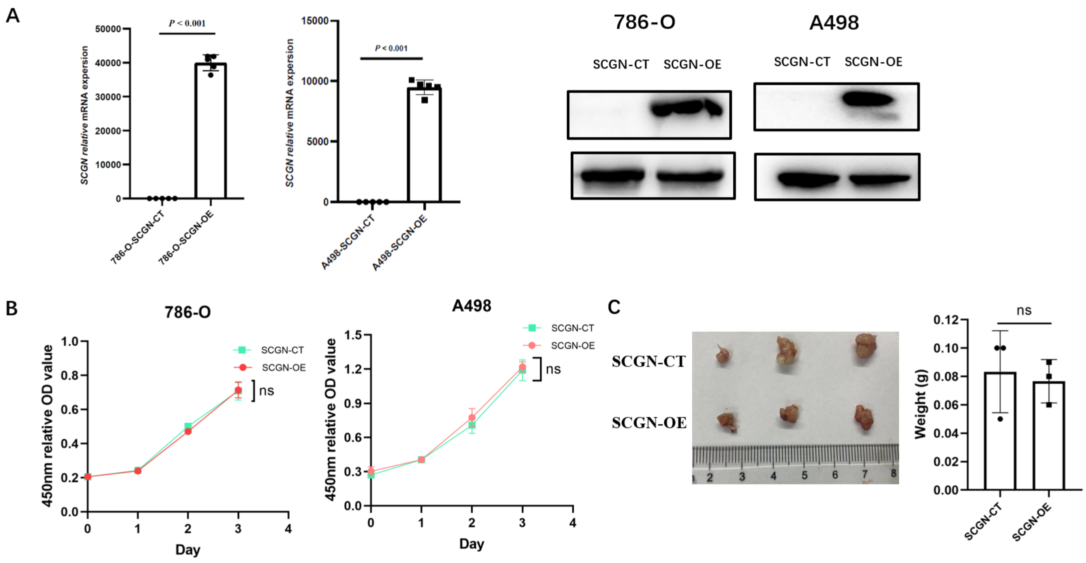

Figure S1. SCGN did not affect the proliferation of ccRCC cells.

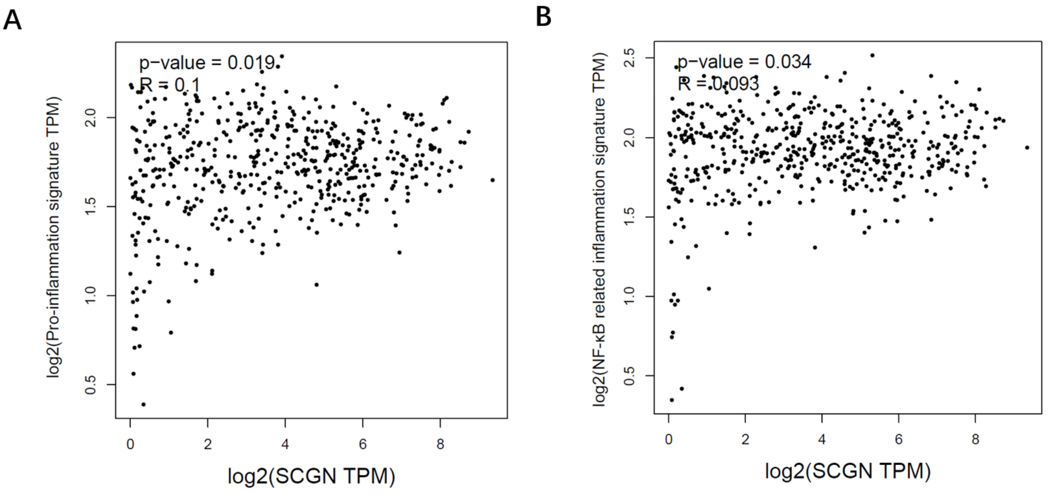

Figure S2. SCGN and inflammation signaling are strongly correlated.

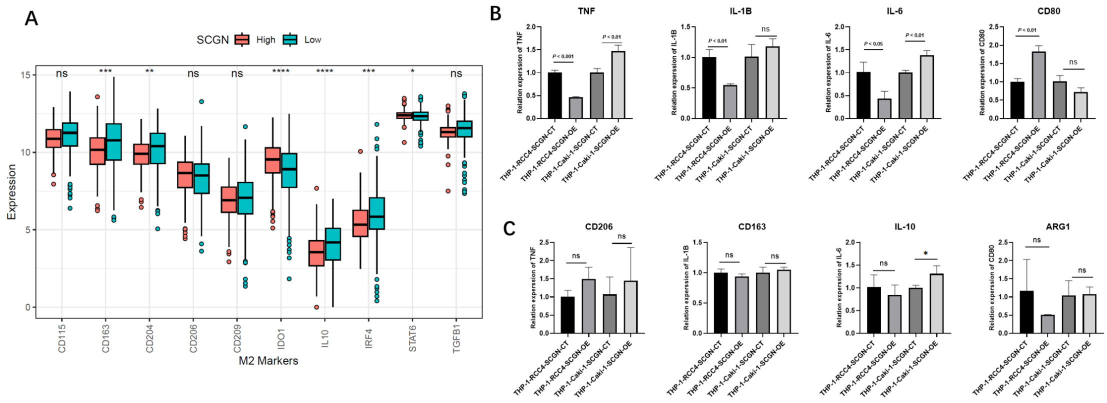

Figure S3. SCGN regulates macrophage infiltration but does not influence polarization.

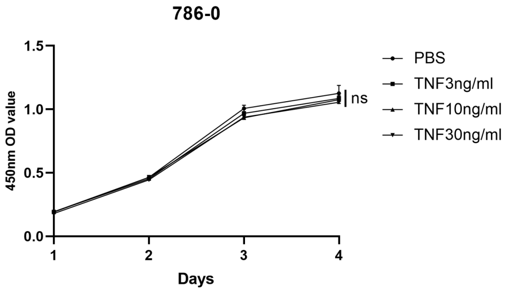

Figure S4. TNF did not affect ccRCC proliferation.

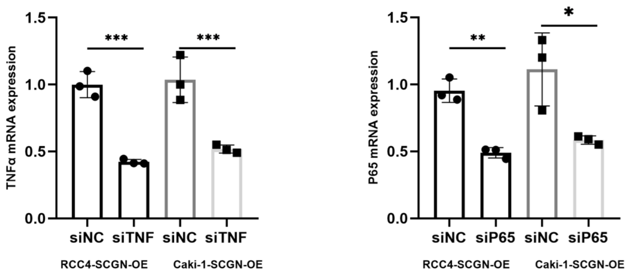

Figure S5. siRNA knockout inefficiency validation.

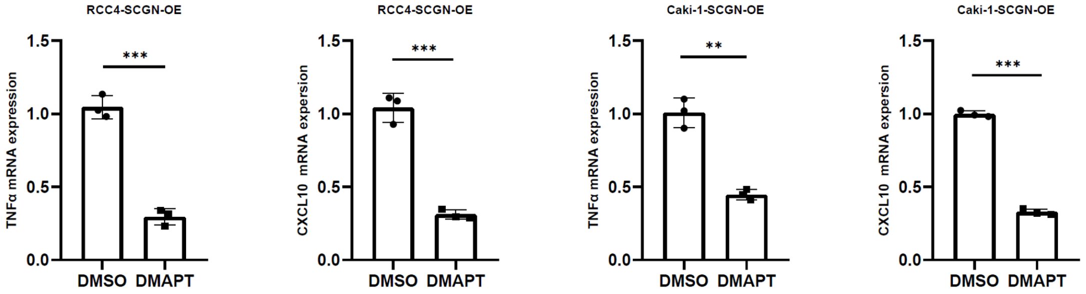

Figure S6. Inhibition of NF-κB reduces cytokine and chemokine expression.

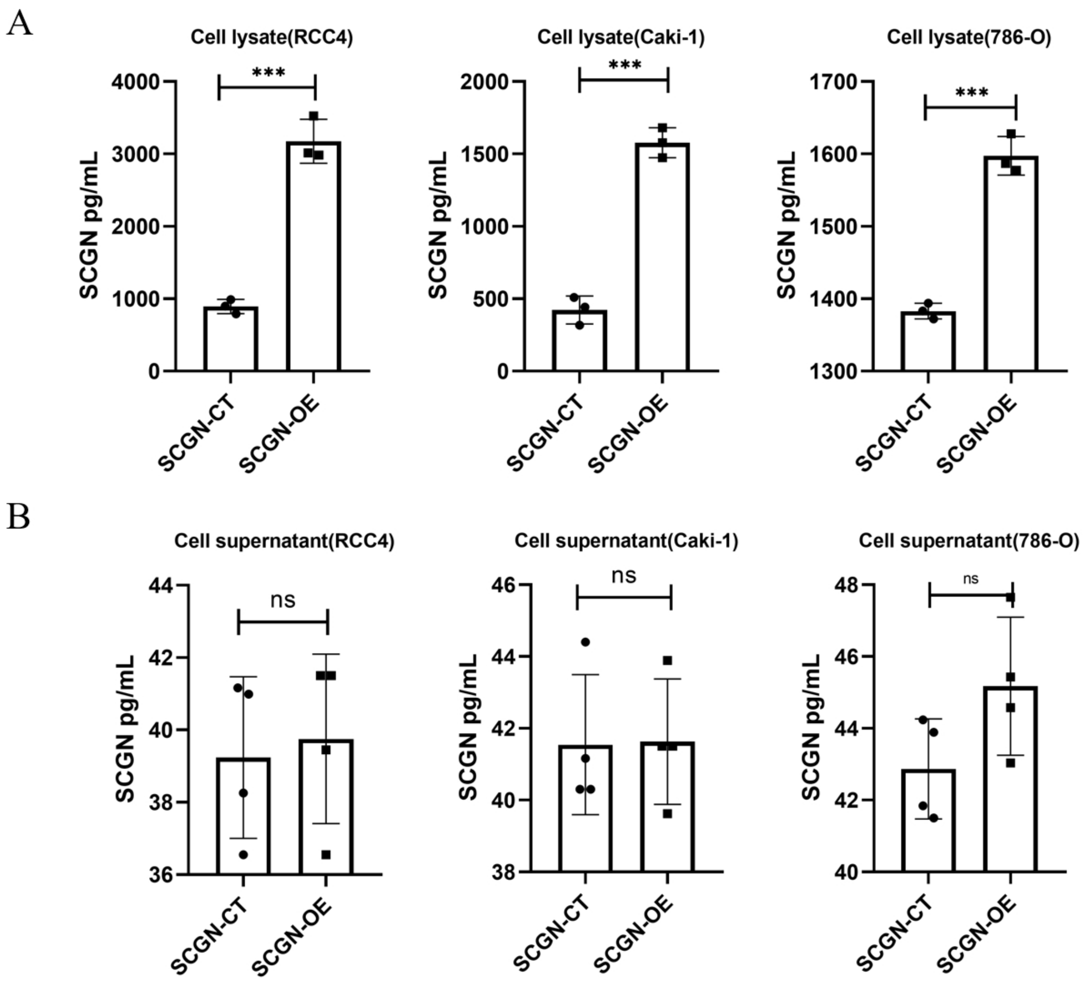

Figure S7. SCGN concentrations in cell lysates and supernatants.

Table S1.

Primer	Sequence(5'-3')
SCGN-F	AACTGGGTACTGATGACACGG
SCGN-R	TCTTTAGAGGCATCTTGGGTAGT
CXCL10-F	GAGCCTACAGCAGAGGAACC
CXCL10-R	GCTGATGCAGGTACAGCGT
ACTB-F	GTTGTCGACGACGAGCG
ACTB-R	GCACAGAGCCTCGCCTT
TNFα-F	CCTCTCTCTAATCAGCCCTCTG

TNFα-R	GAGGACCTGGGAGTAGATGAG
P65-F	ATGTGGAGATCATTGAGCAGC
P65-R	CCTGGTCCTGTGTAGCCATT
IL-1β-F	ATGATGGCTTATTACAGTGGCAA
IL-1β-R	GTCGGAGATTCGTAGCTGGA
IL-6-F	ACTCACCTCTTCAGAACGAATTG
IL-6-R	CCATCTTTGGAAGGTTCAGGTTG
CD80-F	AAACTCGCATCTACTGGCAAA
CD80-R	GGTTCTTGTACTCGGGCCATA
CD206-F	TCCGGGTGCTGTTCTCCTA
CD206-R	CCAGTCTGTTTTTGATGGCACT
CD163-F	TTTGTCAACTTGAGTCCCTTCAC
CD163-R	TCCCGCTACACTTGTTTTCAC
IL-10-F	GACTTTAAGGGTTACCTGGGTTG
IL-10-R	TCACATGCGCCTTGATGTCTG
ARG1-F	GTGGAAACTTGCATGGACAAC
ARG1-R	AATCCTGGCACATCGGGAATC

Table S2.

siRNA	Sequence(5'-3')
TNF	CTGGTATGAGCCCATCTATCTGG
P65	ACCATCAACTATGATGAGTTTCC
